# Markers of Inflammation and Hypofibrinolysis Are Associated with Cognitive Dysfunction and Motor Performances in Atrial Fibrillation Patients on Oral Anticoagulant Therapy: Insights from the Strat-AF Study

**DOI:** 10.3390/biomedicines13040941

**Published:** 2025-04-11

**Authors:** Francesco Alfano, Francesca Cesari, Anna Maria Gori, Martina Berteotti, Emilia Salvadori, Betti Giusti, Alessia Bertelli, Filippo Fratini, Angela Rogolino, Benedetta Formelli, Francesca Pescini, Enrico Fainardi, Eleonora Barucci, Giulia Salti, Arianna Cavaliere, Andrea Ginestroni, Rossella Marcucci, Anna Poggesi

**Affiliations:** 1 Department of Experimental and Clinical Medicine, University of Florence, 50134 Florence, Italy; francesca.cesari@gmail.com (F.C.); annamaria.gori@unifi.it (A.M.G.); martina.berteotti@unifi.it (M.B.); betti.giusti@unifi.it (B.G.); alessia.bertelli@unifi.it (A.B.); rogolinoa@aou-careggi.toscana.it (A.R.); rossella.marcucci@unifi.it (R.M.); 2 Center for Atherothrombotic Diseases, Careggi University Hospital, 50134 Florence, Italy; 3 Department of Biomedical and Clinical Sciences, University of Milan, 20157 Milan, Italy; emilia.salvadori@unimi.it; 4 NEUROFARBA Department, Neuroscience Section, University of Florence, 50134 Florence, Italy; filippo.fratini@unifi.it (F.F.); benedetta.formelli@unifi.it (B.F.); francesca.pescini@unifi.it (F.P.); eleonora.barucci@unifi.it (E.B.); arianna.cavaliere@unifi.it (A.C.); anna.poggesi@unifi.it (A.P.); 5 Stroke Unit, Careggi University Hospital, 50134 Florence, Italy; saltig@aou-careggi.toscana.it; 6 Neuroradiology Unit, Careggi University Hospital, Department of Experimental and Clinical Biomedical Sciences, University of Florence, 50134 Florence, Italy; henryfai@tin.it (E.F.); a.ginestroni@gmail.com (A.G.)

**Keywords:** atrial fibrillation, oral anticoagulant therapy, biomarkers, inflammation, hypofibrinolysis, cognitive performance, motor performance

## Abstract

**Background:** Atrial fibrillation (AF) is the most common supraventricular arrythmia and one of the most commonly encountered heart conditions in clinical practice. Emerging evidence suggests a significant role of inflammation in the pathogenesis of AF. Population studies have also suggested an association between AF and cognitive impairment and dementia. The aim of this study is therefore to assess, in a population of AF patients on oral anticoagulant therapy, the association between circulating biomarkers involved in the pathogenesis of AF and the cognitive and motor performances of the enrolled patients. **Methods:** The Strat-AF study is an observational, prospective, single-center, hospital-based study enrolling elderly patients with AF. Results refer to 180 subjects who underwent a complete clinical, biohumoral, cognitive, and functional evaluation. **Results:** At multivariate logistic regression, Clot Lysis Time (CLT) and circulating levels of von Willebrand Factor (vWF) remained significantly associated with pathological performances at the Stroop test (expressed as execution time) [OR 95% CI 1.54 (1.02–2.35), *p* = 0.042 and 1.75 (1.08–2.82), *p* = 0.023, respectively]. With regard to the Short Physical Performance Battery (SPPB), the circulating levels of IL-8 remained significantly associated with the clinical endpoint [OR 95% CI 2.19 (1.13–4.25), *p* = 0.020]. **Conclusions:** Our results suggest a potential innovative tool able to identify AF patients at risk of worse prognosis in terms of cognitive and motor performances. The clinical relevance of these results is due to the fact that we have no efficient methods to predict a deterioration in the cognitive performance and, consequently, the possible onset of dementia in AF patients undergoing oral anticoagulant therapy.

## 1. Introduction

Atrial fibrillation (AF) is the most common supraventricular arrythmia and one of the most commonly encountered heart conditions in clinical practice, being associated not only with increased risk of stroke and systemic embolism, but also with increased morbidity and mortality. The prevalence of AF is expected to increase significantly in the next few decades due to the aging of the population, an increasing burden of comorbidities, improved awareness, and new technologies for its diagnosis and detection [1].

Emerging evidence suggests a significant role of inflammation in the pathogenesis of AF [2]. Several studies have described an association between AF and abnormal circulating levels of prothrombotic plasma markers, such as fibrinogen, von Willebrand factor (vWF), and soluble P-selectin, suggesting that the arrhythmia itself contributes to the development of a pro-thrombotic state [3]. Moreover, various inflammatory markers and mediators, including C-reactive protein (CRP), tumor necrosis factor alpha (TNF)-alpha, interleukin (IL)-2, IL-6, and IL-8, are associated with the presence of AF and its outcomes [4].

Population studies have also suggested an association between AF and cognitive impairment and dementia. Patients with AF have an increased risk of cognitive impairment and dementia [5,6,7,8,9]. These two clinical conditions share similar risk factors, such as age, diabetes, hypertension, and heart failure, which could confound the association [10]. In addition, stroke, a serious complication of AF, is a well-described risk factor for cognitive impairment [11]. Silent cerebral emboli, small vessel disease (SVD), and chronic hypoperfusion during AF may also represent a plausible pathophysiological link between AF and cognitive impairment. Even when controlled for the confounding effects of stroke, comorbidities, and oral anticoagulant therapy (OAT), AF exposure still remains significantly associated with vascular dementia [12]. In this setting, the timing of OAT initiation seems relevant, as studies have shown a reduced risk of cognitive decline among patients who started an early anticoagulant treatment after the diagnosis of AF, suggesting a possible dose–response effect of unprotected time in AF [7,13,14].

Circulating biomarkers play an important role in the pathophysiological changes underlying AF and have been proposed as useful tools for both risk stratification and prediction of AF progression, having shown to improve the predictive ability of clinical risk scores [15,16,17].

Emerging evidence also suggests a possible relationship between pro-inflammatory cytokines and motor performance in AF patients [18].

The aim of this study is therefore to assess, in a population of AF patients on oral anticoagulant therapy for the primary or secondary prevention of ischemic stroke, the association between circulating biomarkers involved in the pathogenesis of AF the and the cognitive and motor performances.

## 2. Materials and Methods

### 2.1. Study Population

The observational prospective Strat-AF study (Stratification of cerebral bleeding risk in AF) is a single-center, hospital-based study enrolling elderly patients with AF. Patients were referred from the outpatient clinic of Atherothrombotic Disease Center of Careggi University Hospital, where they were followed for the management of OAT in primary or secondary prevention of thromboembolic events. The main aim of the study was to evaluate the relationship between circulating biomarkers involved in the pathogenesis of AF and the cognitive and motor performances of the enrolled patients. For this purpose, patients ≥ 65 years with a diagnosis of AF, ongoing OAT with vitamin K antagonists (VKA) or direct oral anticoagulants (DOACs) and with no contraindications for cerebral magnetic resonance imaging (MRI) were enrolled.

The Strat-AF study was conducted in accordance with the Declaration of Helsinki, and ethical approval was obtained by the Ethics Committee of Careggi University Hospital on 14 March 2017 (project identification code 16RFAP); all participants gave written informed consent for the inclusion before their enrollment. The study design and methodology have been described in a previous paper [19].

For the aim of this study, demographic characteristics (including age, sex, and years of education), previous stroke events, cardiovascular risk factors, and comorbidities (hypertension, diabetes, dyslipidemia, physical activity, smoking habits, alcohol consumption, peripheral arterial disease, ischemic heart disease, myocardial infarction, heart failure) were collected and used for the analysis.

The study protocol was approved by each ethical committee, and all patients gave informed consent.

### 2.2. Laboratory Determinations

For the whole study population, blood samples were collected in the afternoon in tubes without anticoagulant and/or with citrate (3.2%, 0.109 M) and analyzed in a unique central laboratory. Blood samples were centrifuged at room temperature at 1500× *g* for 15 min, and the supernatants were stored in aliquots at −80 °C until the measurement of blood biomarkers, which was performed six months after the enrollment.

The levels of the different inflammatory biomarkers [interleukin (IL)-4, IL-6, IL-8, IL-10, Tumor Necrosis Factor alpha (TNF-alpha), chemokine (C-C motif) ligand 2 (CCL2) also referred to as monocyte chemoattractant protein 1, C-X-C motif chemokine ligand 10 (CXCL10) also known as Interferon gamma-induced protein 10, Intercellular Adhesion Molecule-1 (ICAM-1), Vascular cell adhesion protein 1 (VCAM-1), and Vascular Endothelial Growth Factor (VEGF)] were determined on serum samples using a Bio-Plex suspension array system and R&D Kits (R&D System, Milan, Italy).

Growth/differentiation factor 15 (GDF-15), Proprotein Convertase Subtilisin/Kexin type 9 (PCSK-9) and Soluble Urokinase Plasminogen Activator receptor (s-UPAR) levels were assessed on serum samples by using an automated

ELISA platform (ELLA) was used according to the manufacturer’s instructions (Biotechne, Milan, Italy).

As regards clotting parameters, the activity of VWF and D-Dimer levels were determined on citrated plasmas by a latex particle-enhanced immunoturbidimetric assay (Werfen, Milan, Italy).

PAI-1 Antigen levels were assessed on plasma samples by immunoenzymatic assay (Hyphen Biomed, Neuville-sur-Oise, France).

Thrombin generation was assessed according to the method described by Hemker [20], with a calibrated automated thrombogram (CAT) using a Fluoroskan Ascent^®^ microplate fluorimeter (Thermo Fisher Scientific, Waltham, MA, USA).

The lysis of a tissue-factor-induced clot by exogenous t-PA was assessed by monitoring changes in turbidity during clot formation and subsequent lysis according to Lisman [21], with some changes in t-PA concentrations employed and a clot lysis time (CLT) calculation model.

For each sample, all measures were performed in duplicate.

### 2.3. Cognitive and Functional Protocol

Functional status was assessed using the Activities of Daily Living (ADL) scale (number of preserved items, score range 0–6, with higher scores suggesting less disability) [22] and the Instrumental Activities of Daily Living (IADL) scale (number of impaired items, score range 0–8, with higher scores suggesting more disability) [23], with mood assessment using the Geriatric Depression Scale (GDS) [24] and motor performance using the Short Physical Performance Battery (SPPB) [25].

A multidomain cognitive assessment (including global functioning, attention, speed, and motor control) was administered to each included patient by means of the extensive battery of tests as follows:

- Global cognitive efficiency, by means of the Montreal Cognitive Assessment (MoCA). It is a 10 min cognitive screening tool created to detect mild cognitive impairment (MCI) and suggested from the National Institute of Neurological Disorders and Stroke—Canadian Stroke Network (NINDS-CSN). MoCA was thought to be specifically sensitive to frontal, attention, and executive deficits [26,27,28], as it covers eight cognitive domains (score range 0–30).
- Attention, by means of the Color Word Stroop test [29]. It is a measure of concentration effectiveness and deals with response inhibition and selective attention. The activity required by this test is a selective processing of only one visual feature while continuously blocking out the processing of others. The execution time and the errors committed are recorded.

All raw test scores were demographically corrected according to the Italian population normative data, and their adjusted scores were subsequently recoded as normal, borderline, or abnormal according to equivalent scores (ES) methodology [30,31]. ES methodology is a non-parametric norming method based on percentiles with an ordinal 5-point scale (ranging from 0 to 4). The main purpose of this methodology is to fix the outer tolerance limit of the left queue of the adjusted scores in order to assess—with a known risk of error (<5%)—the cut-off splitting the bottom 5% of the population which represents a pathological performance (ES = 0). An ES = 4 indicates an optimal performance (equal to or better than the median). ES = 1 suggests a borderline performance (an adjusted score between the outer and inner confidence limits for the fifth centile of the normal population), while the remaining ESs (2 and 3) represent normal performances.

### 2.4. Statistical Analysis

As a main explanatory variable, we used the baseline of inflammatory markers, thrombin generation parameters, CLT, D-Dimer, vWF, and PAI-1 antigen levels. Differences in these biomarker values were analyzed in relation to demographic and clinical characteristics and across subgroups of patients according to the outcomes.

We used a Pearson χ^2^ to test for significance while comparing binary variables and ANOVA or Kruskal–Wallis H Test for numeric variables as appropriate. Values are presented as median and interquartile range if they had a non-Gaussian distribution.

To analyze differences in the biomarker levels, we chose the Mann–Whitney U Test. The net effect of each biomarker’s baseline on outcomes was then assessed by a logistic regression model, including as covariates age, sex, CHA2DS2-VASc, years of education, previous stroke, GDS, and oral anticoagulation type (VKA or DOACs).

Receiver-operating characteristic (ROC) curves for the worse lower limbs’ performance (SPPB ≤ 10) of the two models of logistic regression (model 1: age, gender, CHA2DS2-VASc, years of education, previous stroke, GDS, and OAT (VKA or DOACs); model 2: model 1 + IL-8) were calculated. Similarly, we calculated the ROC curves for the pathological performance at the Stroop test (expressed as execution time) by using the model 3: age, gender, CHA2DS2-VASc, years of education, previous stroke, Geriatric Depression Scale, and Oral Anticoagulation type (VKA or DOACs) and model 4: model 3 + VWF and CLT.

We chose variables for the adjustment of multivariate logistic regression analysis according to the significance at univariate analysis.

To correct results for multiple comparisons, we used the false discovery rate testing in all the statistical analyses.

A significant level was defined as *p* < 0.05. All analyses were performed with SPSS 29.0 (SPSS Inc., Chicago, IL, USA) and Stata 13.0 (Lakeway Dr, College Station, TX, USA).

## 3. Results

Results refer to 180 patients (mean age 77.72 ± 6.70 years, females *n* = 67, 37.2%) enrolled in the Strat-AF study with complete clinical, biohumoral, cognitive, and functional evaluation. There is a difference in the number of patients between this manuscript and the work of Salvadori et al. [32] because we were unable to collect sufficient blood samples from two patients to perform the biomarker analysis. Demographic and clinical characteristics are shown in Table 1. All the subjects of the study were on OAT: 31.1% (*n* = 56) were on VKA, and 68.9% (*n* = 124) were on DOACs. In particular, 92.9% (*n* = 52) were on warfarin, and 7.1% (*n* = 4) were on acenocoumarol. Regarding those who were on VKA, 78.8% (*n* = 52) had an adequate time in therapeutic range (TTR) (>60%). With regard to the patients anticoagulated with DOACs, 33.9% (*n* = 42) were on apixaban, 30.6% (*n* = 38) were on dabigatran, 21.0% were on rivaroxaban, and 14.5% (*n* = 18) were on edoxaban. In the DOACs group, we performed the dosage of each type of DOAC (apixaban, dabigatran, edoxaban, rivaroxaban) and in more than 90% of the patients the DOAC concentrations were in line with the average concentration reported in clinical studies.

### 3.1. Circulating Biomarkers According to Cognitive Performance

#### 3.1.1. Geriatric Depression Scale

One hundred and thirty-eight patients (76.7%) obtained normal scores at the GDS (<5 points: no depressive symptoms), 31 patients (17.2%) had mild depressive symptoms (6–9 points), and 11 patients (6.1%) had severe depressive symptoms (>10 points).

We found a significant correlation between the circulating levels of CXCL-10 and the scores of the GDS (ρ = 0.163, *p* = 0.029) (Table 2).

#### 3.1.2. Montreal Cognitive Assessment

At the administration of the Montreal Cognitive Assessment (MoCA), whose medium equivalent score was 2.63 ± 1.37 (median ± DS), there was an inverse correlation between the circulating level of IL-10 and the equivalent scores of the MoCA (ρ = −0.182, *p* = 0.015) (Table 2).

#### 3.1.3. Stroop Test

Stroop test’s medium execution time was 2.78 ± 1.59. Stroop test’s number of error was 3.44 ± 1.06. Both the execution time and the number of errors were expressed as equivalent scores.

At univariate analysis the circulating levels of VCAM-1, vWF, and GDF15 were significantly associated with Stroop test’s execution time as negative correlation (ρ = −0.201, *p* = 0.007; ρ = −0.251, *p* = <0.001 and ρ = −0.171, *p* = 0.027, respectively). In addition, there was a significant inverse correlation between the circulating levels of IL-10 and GDF15 and the errors committed at the Stroop test (ρ = −0.204, *p* = 0.006 and ρ = −0.228, *p* = 0.003, respectively) (Table 2).

Circulating levels of vWF and GDF15 were significantly higher in patients with pathological performances at the Stroop test (expressed as execution time) with respect to patients with normal scores (*p* = 0.001 and *p* = 0.019, respectively). Circulating levels of vWF were also significantly higher in patients with pathological performances at the Stroop test expressed as numbers of error, with respect to patients with normal scores (*p* = 0.007) (Table 3).

#### 3.1.4. ADL and IADL

With regard to the Activities of Daily Living, 141 (78.3%) patients had 6 preserved items, 32 (17.8%) had 5 preserved items, 5 (2.8%) had 4 preserved items, and only 2 (1.1%) had 2 preserved items. Concerning the Instrumental Activities of Daily Living, 81 (45%) patients had no impaired items, 21 (11.7%) had 1 impaired item, 25 (13.9%) had 2 impaired items, 33 (18.3%) had 3 impaired items, 12 (6.7%) had 4 impaired items, 2 (1.1%) had 5 impaired items, 3 (1.7%) had 6 impaired items, 1 (0.6%) had 7 impaired items, and 2 (1.1%) had no preserved items. At the univariate analysis, there was no association between the number of preserved items at the Activities of Daily Living scale and the analyzed circulating biomarkers, but there was a significant association between the circulating levels of PAI-1, GDF15, and PCSK9 and the number of impaired items at the Instrumental Activities of Daily Living scale (ρ = 0.180, *p* = 0.016; ρ = 0.224, *p* = 0.004 and ρ = 0.155, *p* = 0.046, respectively), as shown in Table 4.

#### 3.1.5. Short Physical Performance Battery and Gait Speed Test

SSPB’s medium score was 9.49 ± 2.08. There was an inverse correlation between the circulating level of PAI-1, of vWF, of GDF15, of PCSK9, and of sUPAR and the scores of the SPPB (ρ = −0.326, *p* < 0.001; ρ = −0.210, *p* = 0.005; ρ = −0.218, *p* = 0.005; and ρ = −0.201, *p* = 0.009, respectively), as shown in Table 4.

By dichotomizing the SPPB score on the basis of the normality cut-off (reduced lower libs’ performance if total score < 10 points), the circulating levels of IL-8, PAI-1, vWF, GDF15, and of PCSK9 resulted significantly higher in patients with worse lower libs’ performances, if compared to patients with normal scores (*p* = 0.031, *p* = 0.002, *p* = 0.015, *p* = 0.028, and *p* = 0.041, respectively), as shown in Table 5.

The third subtest of the SPPB (the four-meter gait speed) analyzes the gait, both as speed and as execution time. There was an inverse correlation between the circulating level of PAI-1, GDF15, sUPAR, and PCSK9 and the gait speed (ρ = −0.205, *p* = 0.006; ρ = −0.232, *p* = 0.003; ρ = −0.178, *p* = 0.022; and ρ = −0.256, *p* < 0.001, respectively). Conversely, there was a positive correlation between the circulating level of PAI-1, GDF15, sUPAR, and PCSK9 and the gait speed (ρ = 0.205, *p* = 0.006; ρ = 0.232, *p* = 0.003; ρ = 0.178, *p* = 0.022; and ρ = 0.256, *p* < 0.001, respectively) (Table 4).

By dividing the gait speed on the basis of the normality cut-off (pathological if <5 s), the circulating levels of PAI-1 and of sUPAR resulted significantly higher in patients with pathological performances at the gait speed test when compared to patients with normal scores (*p* = 0.046 and *p* = 0.015, respectively) (Table 5).

### 3.2. Multivariate Analysis

In order to establish an independent association between circulating biomarkers and the cognitive and motor performances, we performed a multivariate logistic regression analysis adjusted for age, sex, CHA2DS2-VASc, years of education, previous stroke, Geriatric Depression Scale, and oral anticoagulation type (VKA or DOACs).

At multivariate logistic regression, the CLT and vWF circulating levels remained significantly associated with pathological performances at the Stroop test (expressed as execution time) [OR 95% CI 1.54 (1.02–2.35), *p* = 0.042 and 1.75 (1.08–2.82), *p* = 0.023, respectively].

With regard to SPPB, the circulating levels of IL-8 remained significantly associated with the clinical endpoint [OR 95% CI 2.19 (1.13–4.25), *p* = 0.020].

ROC analyses demonstrated that the addition of IL-8 (model 2) to a model that included factors known to affect the outcome SPPB ≤ 10 (model 1) significantly improved the area under the curve (AUC) for the prediction of worse lower limbs performance [model 1: AUC  =  0.72 (95% CI 0.64–0.80); model 2: AUC  =  0.81 (95% CI 0.74–0.88), *p*  =  0.015]. Furthermore, the addition of vWF and CLT (model 4) to a model that included factors known to affect the pathological performance at the Stroop test (express as execution time—model 3) significantly improved the area under the curve for the prediction of worse Stroop test execution time [model 3: AUC  =  0.74 (95% CI 0.66–0.82); model 4: AUC  =  0.80 (95% CI 0.72–0.87), *p*  =  0.047].

## 4. Discussion

We found that markers of endothelial dysfunction and inflammation (vWF and IL-8) as well as hypofibrinolysis (documented by prolonged CLT) are significantly associated with cognitive performance in patients with AF patients on oral anticoagulant therapy for the primary or secondary prevention of thromboembolic events.

The aim of the present study was to investigate a possible relationship between some circulating biomarkers involved in the pathogenesis of AF and the cognitive and motor performances of the enrolled patients. For the purpose of the study, we took into account different circulating biomarkers involved in pathways, such as inflammation and hemostasis, correlating these parameters with the equivalent scores of some neuropsychological test and with some indexes of motor performance.

It is known that vWF plays an important role in the molecular mechanisms underlying the pathogenesis of AF and its plasma levels may improve thrombosis risk stratification in AF patients [33]. Hemostasis and inflammation are complex and interrelated processes that are also associated with a variety of phenotypes, but there is limited case–control evidence associating markers of hemostasis and inflammation with dementia [33,34,35,36].

Gallacher and colleagues presented data on 865 men free of vascular disease, with biomarker measurements, whose hemostasis factors were measured at the age of 45 to 59 years, and cognition and dementia were determined up to the age of 65 to 84 years. During 17 years of follow-up, 59 of the men developed dementia, and 112 developed cognitive impairment/no dementia. Vascular dementia appeared to be related to certain coagulation pathways, in particular accentuated clot formation and fibrin plug formation [37]. Quinn and colleagues also described an association between activation of hemostasis (in particular, endothelial dysfunction) and vascular dementia [38].

vWF has also been associated with markers of cerebral small-vessel (SVD) disease, which is a well-known risk factor for dementia. In this setting, chronic cerebral ischemia could further link vWF to cognitive decline via (covert) brain infarcts or cortical micro-infarcts [39]. Our findings may therefore serve as generators of hypothesis, suggesting a relationship between small vessel disease and subsequent pathological cognitive performances, as an epiphenomenon of an endothelial dysfunction [40,41,42].

Our results show that even an impaired fibrinolysis (as suggested by the prolonged CLT) may play a role in reducing the cognitive performance of AF patients. In the literature, there are some data according to which CLT seems to predict stroke during anticoagulant therapy in patients with AF and that patients with permanent AF and previous stroke are characterized by prolonged CLT if compared to those without stroke [43,44].

Moreover, this is the first time that a link between IL-8 circulating levels and motor performance is reported [45], but there is growing evidence that suggests a possible role of IL-8 in Alzheimer’s disease (AD) pathogenesis, as IL-8 is usually upregulated in AD, favoring neuroinflammation [46,47,48,49].

Our data suggest that, in clinical practice, through venous blood samples obtained at the beginning of oral anticoagulation in AF patients, we could identify those at high risk of worse cognitive performance and impaired motor performances by means of a biomarker screening. Moreover, a cluster of interrelated biomarkers, also associated with cerebral SVD, may better explain the underlying pathological processes with respect to single biomarkers.

However, our study has some limitations. Firstly, the patients were carefully selected according to the inclusion criteria of the study and the relatively small sample size of the population posed some limitations to our study’s results. A larger cohort would increase the statistical power of the analysis and allow for detection of a more subtle relationship between variables. Secondly, lifestyle factors, such as diet, sleep, and physical activity levels, could impact both systemic inflammation and cognitive/motor performance. Unfortunately, we collected data regarding diet, sleep patterns, and physical activities only in few patients, so we were not able to evaluate their role in systemic inflammation and cognitive/motor performance. Lastly, the cross-sectional design of the present study does not allow us to identify those patients at higher risk of developing a worsening of their cognitive or motor performance according to laboratory parameters, considering that we only took a picture of the baseline biohumoral profile. Therefore, we are not able to establish a cause–effect relationship between the circulating levels of the analyzed biomarkers and the longitudinal development of alterations in the cognitive or motor performances of the enrolled patients. A future approach could include long-term follow-up to assess cognitive and motor performance progression over time.

## 5. Conclusions

Our results suggest a potential innovative tool able to identify AF patients at risk of worse prognosis in terms of cognitive and motor performances. The clinical relevance of these results is due to the fact that we have no efficient methods to predict a deterioration in the cognitive performance and, consequently, the possible onset of dementia in AF patients undergoing oral anticoagulant therapy.

## Figures and Tables

**Table 1:** Demographic and clinical characteristics of the baseline Strat-AF study cohort (*n* = 180). Results are expressed as mean ± DS and as percentage.

Demographic and Clinical Characteristics	Total Cohort (*n* = 180)
Age [yrs], (mean ± SD)	77.72 ± 6.70
Female sex, *n* (%)	67 (37.2%)
Schooling [yrs], (mean ± SD)	9.36 ± 4.27
Stroke, *n* (%)	38 (21.1%)
Coronary artery disease, *n* (%)	19 (10.6%)
Heart failure, *n* (%)	24 (13.3%)
Peripheral arterial pathology, *n* (%)	14 (7.8%)
Hypertension, *n* (%)	147 (81.7%)
Diabetes, *n* (%)	23 (12.8%)
Dyslipidaemia, *n* (%)	92 (51.1%)
Physical activity (lack of), *n* (%)	116 (64.4%)
Smoke, *n* (%)	105 (58.3%)
Alcohol consumption, *n* (%)	95 (52.8%)
BMI [kg/m^2^], (mean ± SD)	26.40 ± 3.79
CHA_2_DS_2_-VASc Score (mean ± SD)	3.71 ± 1.41
HAS-BLED (mean ± SD)	1.82 ± 0.87

**Table 2:** Associations between circulating biomarkers and GDS, MoCA, and Stroop test.

Biological Markes	GDS (*n* = 180)		MoCA E.S. (*n* = 180)		Stroop Test			
					Time of Execution, E.S. (*n* = 180)		Number of Errors (*n* = 180)	
	Rho	*p*-Value	Rho	*p*-Value	Rho	*p*-Value	Rho	*p*-Value
IL-4	−0.134	0.072	0.021	0.782	−0.034	0.653	−0.040	0.596
IL-6	0.100	0.172	−0.068	0.351	−0.128	0.087	−0.026	0.734
IL-8	0.045	0.550	0.099	0.185	−0.061	0.419	−0.051	0.494
IL-10	0.061	0.416	**−0.182**	**0.015**	−0.115	0.123	**−0.204**	**0.006**
TNFα	0.019	0.795	−0.038	0.614	−0.084	0.261	0.003	0.963
CCL-2	−0.136	0.069	−0.103	0.169	0.034	0.650	0.093	0.213
CXCL-10	**0.163**	**0.029**	−0.036	0.631	−0.100	0.182	−0.011	0.881
ICAM-1	−0.101	0.178	−0.009	0.908	−0.116	0.121	−0.037	0.618
VCAM-1	0.093	0.214	−0.051	0.500	**−0.201**	**0.007**	−0.146	0.051
VEGF	−0.094	0.209	−0.014	0.857	−0.055	0.464	0.067	0.368
PAI-1	0.004	0.957	−0.088	0.242	−0.054	0.478	−0.095	0.210
vWF	0.043	0.569	0.036	0.636	**−0.251**	**<0.001**	−0.081	0.284
Lag time	−0.024	0.747	−0.046	0.538	−0.004	0.957	−0.041	0.590
Peak	−0.059	0.420	0.018	0.810	−0.034	0.658	−0.006	0.935
Time to peak	0.029	0.697	−0.075	0.307	0.014	0.853	−0.005	0.947
ETP TM-	0.022	0.763	−0.024	0.751	−0.014	0.850	−0.005	0.945
ETP TM+	−0.055	0.451	0.023	0.756	−0.032	0.674	0.007	0.929
ETP ratio	−0.042	0.569	0.011	0.883	−0.061	0.417	−0.010	0.897
Clot lysis time	0.044	0.567	−0.089	0.249	−0.097	0.208	−0.057	0.459
D-Dimer	−0.088	0.266	0.038	0.636	−0.018	0.820	−0.043	0.589
GDF15	0.043	0.585	−0.045	0.559	**−0.171**	**0.027**	**−0.228**	**0.003**
PCSK9	−0.092	0.240	−0.115	0.139	−0.105	0.177	−0.015	0.846
sUPAR	−0.049	0.531	−0.018	0.817	−0.101	0.193	−0.019	0.804

**Table 3:** Circulating biomarkers in relation with Stroop test (time of execution and number of errors, dichotomous). Results are expressed as median (range).

Biological Markes	Stroop Test					
	Time of Execution			Number of Errors		
	E.S. 0–1 (*n* = 44)	E.S. 2–4 (*n* = 136)	*p*	E.S. 0–1 (*n* = 44)	E.S. 2–4 (*n* = 136)	*p*
IL-4 [pg/mL], median (IQR)	12.81 (2.54–36.25)	6.70 (4.93–25.38)	0.547	21.65 (5.05–40.28)	6.70 (4.90–25.38)	0.216
IL-6 [pg/mL], median (IQR)	1.79 (0.38–3.71)	1.56 (0.30–2.99)	0.235	2.60 (1.60–5.52)	1.56 (0.30–3.21)	0.107
IL-8 [pg/mL], median (IQR)	9.85 (6.71–16.82)	8.60 (5.43–12.94)	0.118	12.62 (8.08–21.30)	8.73 (5.43–12.77)	0.064
IL-10 [pg/mL], median (IQR)	3.00 (0.32–3.72)	2.89 (5.43–12.94)	0.344	3.46 (0.99–3.72)	2.89 (0.30–3.55)	0.293
TNFα [pg/mL], median (IQR)	2.02 (0.62–5.00)	2.13 (1.06–3.51)	0.798	2.53 (0.81–4.88)	2.04 (0.83–4.00)	0.479
CCL-2 [pg/mL], median (IQR)	320.88 (229.38–475.00)	321.44 (229.34–413.54)	0.991	300.40 (234.03–370.93)	320.88 (228.94–431.60)	0.709
CXCL-10 [pg/mL], median (IQR)	16.21 (11.90–28.07)	14.65 (9.98–23.55)	0.184	18.82 (11.97–32.41)	15.04 (10.49–23.66)	0.240
ICAM-1 [ng/mL], median (IQR)	355.17 (268.45–609.26)	320.39 (251.20–438.35)	0.153	421.36 (279.32–715.25)	326.33 (251.38–444.99)	0.201
VCAM-1 [ng/mL], median (IQR)	1675.60 (1186.90–2333.95)	1337.75 (1004.25–1946.28)	0.058	**2035.30 (1592.20–2300.43)**	**1350.00 (1004.25–1950.90)**	**0.029**
VEGF [pg/mL], median (IQR)	74.06 (47.80–110.79)	64.06 (32.93–108.59)	0.104	57.93 (46.01–97.08)	65.91 (36.88–110.26)	0.812
PAI-1 [ng/mL], median (IQR)	9.93 (6.97–16.73)	8.61 (7.00–13.26)	0.337	10.10 (7.58–19.78)	8.81 (6.97–15.16)	0.347
vWF [%], median (IQR)	**197.30 (158.00–221.70)**	**153.40 (126.40–204.58)**	**0.001**	**220.85 (175.85–231.63)**	**163.90 (130.30–204.50)**	**0.007**
Lag time [min], median (IQR)	9.00 (4.30–54.03)	8.30 (3.30–17.30)	0.378	7.65 (4.40–78.08)	8.30 (3.30–18.35)	0.815
Peak [nM], median (IQR)	47.00 (1.00–195.00)	47.00 (12.83–152.28)	0.977	37.00 (2.90–145.10)	47.00 (11.15–159.55)	0.521
Time to peak [min], median (IQR)	15.70 (8.60–100.00)	14.50 (8.60–28.48)	0.497	19.85 (9.90–82.33)	14.70 (8.30–29.50)	0.417
ETP TM- [nM/min], median (IQR)	599.00 (48.25–1582.70)	548.00 (248.90–1461.00)	0.828	490.90 (61.75–1495.20)	588.00 (214.50–1479.00)	0.664
ETP TM+ [nM/min], median (IQR)	391.00 (1.00–922.00)	339.00 (116.75–1160.75)	0.971	286.50 (35.00–1220.00)	341.00 (116.50–1096.40)	0.721
ETP ratio [ratio], median (IQR)	0.58 (0.01–0.84)	0.67 (0.28–0.91)	0.598	0.73 (0.04–1.06)	0.65 (0.26–0.90)	0.725
Clot lysis time [min], median (IQR)	56.44 (44.90–74.58)	51.60 (40.91–66.09)	0.110	56.45 (47.76–74.10)	52.36 (41.60–67.91)	0.333
D-Dimer [ng/mL], median (IQR)	349 (206–634)	294 (202–508)	0.307	521 (216–3958)	297 (203–528)	0.114
GDF15 [ng/mL], median (IQR)	**192.50 (156.82–284.77)**	**164.0 (123.5–235.6)**	**0.019**	1.97 (1.87–2.83)	1.68 (1.28–2.42)	0.054
PCSK9 [ng/mL], median (IQR)	270.24 (210.94–352.64)	239.56 (197.57–313.44)	0.086	298.09 (211.46–384.75)	244.65 (198.30–325.29)	0.220

**Table 4:** Associations between circulating biomarkers and ADL, IADL, SPPB, and four-meter gait speed test.

Biological Markes	ADL (*n* = 180)		IADL (*n* = 180)		SPPB (*n* = 180)		4 m Gait Speed Test (*n* = 180)	
	Rho	*p*-Value	Rho	*p*-Value	Rho	*p*-Value	Rho	*p*-Value
IL-4	−0.018	0.806	0.041	0.585	0.042	0.574	0.087	0.249
IL-6	0.009	0.908	−0.012	0.874	−0.025	0.737	0.007	0.922
IL-8	−0.109	0.145	0.023	0.755	−0.113	0.13	−0.04	0.592
IL-10	0.063	0.399	−0.044	0.558	0.048	0.524	−0.04	0.598
TNFα	−0.004	0.956	−0.013	0.86	0.004	0.961	0.07	0.34
CCL-2	0.073	0.329	0.089	0.234	0.053	0.477	0.05	0.505
CXCL-10	0.001	0.987	0.055	0.46	−0.087	0.245	−0.146	0.051
ICAM-1	−0.018	0.809	−0.035	0.641	0.021	0.784	0.075	0.318
VCAM-1	−0.014	0.849	−0.035	0.64	0.058	0.436	0.071	0.343
VEGF	−0.137	0.067	0.098	0.19	−0.074	0.322	−0.005	0.944
PAI-1	0.021	0.783	**0.18**	**0.016**	**−0.326**	**<0.001**	**−0.205**	**0.006**
vWF	0.044	0.559	0.036	0.637	**−0.21**	**0.005**	−0.105	0.167
Lag time	0.042	0.587	0.024	0.758	0.003	0.964	0.066	0.388
Peak	−0.019	0.803	0.002	0.977	0.033	0.667	−0.038	0.614
Time to peak	0.032	0.676	0.016	0.829	0.001	0.994	0.042	0.583
ETP TM-	−0.031	0.69	−0.037	0.632	0.04	0.599	−0.042	0.589
ETP TM+	−0.031	0.684	−0.02	0.795	0.055	0.466	−0.038	0.617
ETP ratio	−0.025	0.745	0.018	0.808	0.001	0.993	−0.103	0.172
Clot lysis time	−0.09	0.245	−0.019	0.805	0.008	0.92	−0.041	0.601
D-Dimer	0.152	0.055	0.024	0.759	−0.054	0.495	−0.135	0.09
GDF15	−0.083	0.288	**0.224**	**0.004**	**−0.218**	**0.005**	**−0.232**	**0.003**
PCSK9	−0.003	0.967	**0.155**	**0.046**	**−0.162**	**0.037**	**−0.178**	**0.022**
sUPAR	−0.038	0.630	0.094	0.225	**−0.201**	**0.009**	**−0.256**	**<0.001**

**Table 5:** Circulating biomarkers in relation with dichotomous SPPB and four-meter gait speed test. Results are expressed as median (range).

Biological Markes	Dichotomous SPPB			4 m Gait Speed Test		
	SPPB ≤ 10 (*n* = 113)	SPPB Normal (*n* = 67)	*p*	Compromised (>5 s) (*n* = 113)	Normal (<5 s) (*n* = 67)	*p*
IL-4 [pg/mL], median (IQR)	6.70 (4.60–29.94)	10.80 (5.00–29.40)	0.942	6.10 (2.54–22.04)	10.80 (5.00–32.69)	0.139
IL-6 [pg/mL], median (IQR)	1.56 (0.30–3.51)	1.73 (0.45–2.91)	0.805	1.56 (0.30–4.27)	1.73 (0.38–3.07)	0.781
IL-8 [pg/mL], median (IQR)	**9.14 (6.06–13.70)**	**7.23 (3.41–12.10)**	**0.031**	8.73 (6.74–12.20)	8.88 (5.01–13.15)	0.414
IL-10 [pg/mL], median (IQR)	2.89 (0.30–3.54)	2.89 (0.31–3.56)	0.561	2.89 (1.31–3.46)	2.89 (0.30–3.56)	0.610
TNFα [pg/mL], median (IQR)	2.03 (0.91–3.51)	2.53 (0.73–4.25)	0.573	2.00 (1.06–3.39)	2.30 (0.73–4.25)	0.434
CCL-2 [pg/mL], median (IQR)	325.47 (229.84–405.43)	317.15 (221.48–441.34)	0.970	338.18 (217.80–399.19)	317.15 (230.45–437.56)	0.837
CXCL-10 [pg/mL], median (IQR)	15.04 (11.00–24.01)	16.16 (9.96–23.19)	0.888	15.42 (11.77–24.03)	15.04 (10.16–23.88)	0.564
ICAM-1 [ng/mL], median (IQR)	319.52 (251.53–461.49)	335.66 (255.22–480.73)	0.832	284.84 (246.56–384.34)	332.93 (270.52–480.73)	0.148
VCAM-1 [ng/mL], median (IQR)	1350.00 (1036.30–1942.55)	1518.80 (996.15–2121.50)	0.593	1255.00 (1033.43–1975.63)	1445.90 (1000.00–2078.10)	0.479
VEGF [pg/mL], median (IQR)	61.58 (41.44–103.07)	70.24 (32.18–112.14)	0.854	64.97 (38.60–91.18)	65.65 (36.88–112.14)	0.663
PAI-1 [ng/mL], median (IQR)	**10.57 (7.43–16.74)**	**7.99 (6.39–10.77)**	**0.002**	**12.44 (7.36–19.53)**	**8.60 (6.89–12.27)**	**0.046**
vWF [%], median (IQR)	**191.00 (137.23–217.83)**	**150.10 (121.35)**	**0.015**	183.30 (137.50–217.05)	171.85 (128.60–206.15)	0.320
Lag time [min], median (IQR)	8.30 (3.30–17.35)	8.30 (3.78–19.70)	0.792	8.30 (3.30–14.35)	8.60 (3.60–23.70)	0.473
Peak [nM], median (IQR)	48.00 (10.85–159.78)	45.00 (6.00–161.15)	0.649	40.25 (15.95–155.00)	48.00 (6.50–160.60)	0.921
Time to peak [min], median (IQR)	14.70 (8.30–32.18)	15.60 (9.00–26.15)	0.859	14.65 (9.08–27.98)	14.85 (8.38–34.28)	0.828
ETP TM- [nM/min], median (IQR)	588.00 (247.50–1396.50)	510.50 (142.50–1521.75)	0.717	536.00 (257.60–1310.00)	588.00 (140.00–1649.00)	0.924
ETP TM+ [nM/min], median (IQR)	348.50 (134.00–1048.00)	338.00 (95.25–1210.30)	0.905	376.75 (181.50–844.45)	339.00 (95.13–1174.25)	0.902
ETP ratio [ratio], median (IQR)	0.65 (0.21–0.90)	0.65 (0.29–0.92)	0.776	0.77 (0.32–0.94)	0.62 (0.22–0.84)	0.096
Clot lysis time [min], median (IQR)	53.13 (42.61–68.07)	53.64 (41.03–68.76)	0.664	53.13 (45.12–64.91)	52.93 (41.47–68.90)	0.826
D-Dimer [ng/mL], median (IQR)	316.50 (210.50–604.25)	286.50 (169.25–522.75)	0.222	314.50 (205.50–528.25)	291.00 (205.00–601.00)	0.838
GDF15 [ng/mL], median (IQR)	**1.81 (1.45–2.61)**	**1.59 (1.16–2.22)**	**0.028**	2.08 (1.53–2.62)	1.66 (1.25–2.35)	0.061
PCSK9 [ng/mL], median (IQR)	**258.70 (206.48–351.44)**	**234.70 (189.50–288.15)**	**0.041**	258.70 (213.55–342.87)	243.39 (193.70–313.44)	0.232
sUPAR [ng/mL], median (IQR)	2.97 (2.26–3.74)	2.71 (2.20–3.65)	0.192	**3.35 (2.60–4.21)**	**2.83 (2.17–3.62)**	**0.015**

## Data Availability

The data presented in this study are available on request from the corresponding author. The data are not publicly available due to privacy or ethical restrictions.
